# Functional regulation of Q by microRNA172 and transcriptional co‐repressor TOPLESS in controlling bread wheat spikelet density

**DOI:** 10.1111/pbi.12790

**Published:** 2017-08-09

**Authors:** Pan Liu, Jie Liu, Huixue Dong, Jiaqiang Sun

**Affiliations:** ^1^ National Key Facility for Crop Gene Resources and Genetic Improvement Institute of Crop Science Chinese Academy of Agricultural Sciences Beijing China

**Keywords:** wheat, spike architecture, miR172, *Q* gene, transcriptional repression

## Abstract

Bread wheat (*Triticum aestivum*) spike architecture is an important agronomic trait. The *Q* gene plays a key role in the domestication of bread wheat spike architecture. However, the regulatory mechanisms of Q expression and transcriptional activity remain largely unknown. In this study, we show that overexpression of bread wheat tae‐miR172 caused a speltoid‐like spike phenotype, reminiscent of that in wheat plants with the *q* gene. The reduction in *Q* transcript levels in the tae‐miR172 overexpression transgenic bread wheat lines suggests that the *Q* expression can be suppressed by tae‐miR172 in bread wheat. Indeed, our RACE analyses confirmed that the *Q* mRNA is targeted by tae‐miR172 for cleavage. According to our analyses, the Q protein is localized in nucleus and confers transcriptional repression activity. Meanwhile, the Q protein could physically interact with the bread wheat transcriptional co‐repressor TOPLESS (TaTPL). Specifically, the N‐terminal ethylene‐responsive element binding factor‐associated amphiphilic repression (EAR) (LDLNVE) motif but not the C‐terminal EAR (LDLDLR) motif of Q protein mediates its interaction with the CTLH motif of TaTPL. Moreover, we show that the N‐terminal EAR motif of Q protein is also essentially required for the transcriptional repression activity of Q protein. Taken together, we reveal the functional regulation of Q protein by tae‐miR172 and transcriptional co‐repressor TaTPL in controlling the bread wheat spike architecture.

## Introduction

MicroRNAs (miRNAs) are small noncoding RNAs of 21–24 nucleotides with a wide distribution in animals and plants (Bartel, 2009). Plant miRNAs show a high degree of sequence complementarity to, and are believed to guide the cleavage and/or translational suppression of their target messenger RNAs (Bartel, 2009; Rubio‐Somoza and Weigel, 2011). Previous studies have shown that miRNAs play diverse roles in different processes of plant growth and development, including shoot meristem regulation, leaf development, floral transition and so on (Chen, 2009; Li and Zhang, 2016).

Among numerous plant miRNAs, miR172 is a well‐studied miRNA. Previous studies have shown that miR172 plays important biological roles in different biological processes, especially in floral control (Aukerman and Sakai, 2003; Chen, 2004; Lauter *et al*., 2005; Mathieu *et al*., 2009; Wang *et al*., 2014). For instance, in *Arabidopsis*, miR172 promotes flowering mainly through the suppression of some floral repressor genes, that is a small subfamily of *APETALA2* (*AP2*)‐like transcription factor genes including *AP2*,* TARGET OF EAT1* (*TOE1*) and *TOE2* (Aukerman and Sakai, 2003; Chen, 2004; Jung *et al*., 2007). Studies in maize (*Zea mays*) show that miR172, targeting to the *AP2*‐like downstream gene *INDETERMINATE SPIKELET1* (*IDS1*), regulates sex determination in the floral meristem, as the miR172 loss‐of‐function mutant *ts4* displays feminization of the tassel and increased meristem branching due to the elevated amounts of IDS1 (Chuck *et al*., 1998, 2007). However, the potential roles of miR172 in the regulation of bread wheat (*Triticum aestivum*) development, especially spike morphology, remain to be determined.

The widely cultivated polyploid bread wheat (2*n* = 6*x* = 42, AABBDD) is derived from the domestication processes, which had been important for the agricultural revolution and the establishment of human civilization. The *Q* gene on the long arm of wheat chromosome 5A (*5AQ*), encoding a member of the AP2 family of transcription factors, plays an important role in the domestication of spike architecture. Previous studies have revealed that *Q*, derived from a spontaneous mutation from *q* in allopolyploid wheat, is an important domestication gene that is largely responsible for the domestication‐related traits, such as rachis length, spike shape, free‐threshing character and spike compactness (Faris and Gill, 2002; Muramatsu, 1963; Simons *et al*., 2006; Zhang *et al*., 2011). In contrast, the *q* allele in wild wheat confers a speltoid spike phenotype characterized by a lax head with elongated rachis and non‐free‐threshing seed, and this spike type may have lower crop yield and not allow for the efficient harvest (Faris and Gill, 2002; Muramatsu, 1963; Simons *et al*., 2006; Zhang *et al*., 2011). It is believed that mutation from *q* to *Q* is a profound domestication event which led to the subcompact spike shape and free‐threshing seeds in polyploid wheat and as a result promoted a rapid spread of the wheat cultivation around the world (Zhang *et al*., 2011). However, despite the important role of *Q* gene in the domestication of wheat spike, little is known about the underlying molecular mechanisms of *Q* gene in the regulation of bread wheat spike architecture.

Gene expression is regulated by gene‐specific transcriptional activators and repressors through interaction with general co‐activators and co‐repressors. TOPLESS (TPL) and TOPLESS‐related (TPR) proteins comprise a conserved family of plant transcriptional co‐repressors (Causier *et al*., 2012). TPL proteins have a highly conserved N‐terminal domain containing a Lissencephaly Homologous (LiSH) dimerization motif and a C‐Terminal to LiSH (CTLH) motif (Szemenyei *et al*., 2008). In plants, TPL/TPR co‐repressors regulate development and hormone signalling through interaction with ethylene‐responsive element binding factor‐associated amphiphilic repression (EAR) motifs found in diverse transcriptional repressors (Causier *et al*., 2012). In particular, previous studies showed that TPL‐like proteins in maize and rice are both essential for appropriate establishment of spike/panicle architecture (Gallavotti *et al*., 2010; Yoshida *et al*., 2012). *RAMOSA1 ENHANCER LOCUS2* (*REL2*) gene in maize encodes the TPL‐like transcriptional co‐repressor, and *rel2* mutations increased the inflorescence primary branching phenotype of *ramosa1* (*ra1*) and *ramosa2* (*ra2*) (Gallavotti *et al*., 2010). Accordingly, REL2 is considered to participate in the determinacy of spikelet‐pair meristems in maize inflorescence development through repressing the downstream genes expression (Gallavotti *et al*., 2010). In rice, the *aberrant spikelets and panicle1* (*asp1*) mutant showed reduced number of panicle branches. In fact, *ASP1* encodes a TPL‐related protein in rice, suggesting that TPL should be crucial in the regulation of rice panicle architecture (Yoshida *et al*., 2012).

In this study, we generated transgenic bread wheat plants, in which the *tae‐MIR172* precursor was overexpressed, to characterize the biological function of tae‐miR172. We showed that overexpression of tae‐miR172 caused a speltoid spike phenotype, reminiscent of the wheat with the *q* gene. Further analyses confirmed that the domestication gene *Q* was indeed the downstream target of miR172, and its expression was significantly suppressed by overexpression of miR172. Our results suggest that the Q protein is localized in nucleus and has transcriptional repression activity. Furthermore, we demonstrated that the Q protein could physically interact with the transcriptional co‐repressor TaTPL and this interaction may be required for the transcriptional repression activity of Q protein. In brief, we report the functional regulation of Q protein by tae‐miR172 and transcriptional co‐repressor TaTPL in controlling bread wheat spike architecture variation.

## Results

### Identification of a *tae‐MIR172* precursor in bread wheat

**Figure 1:**
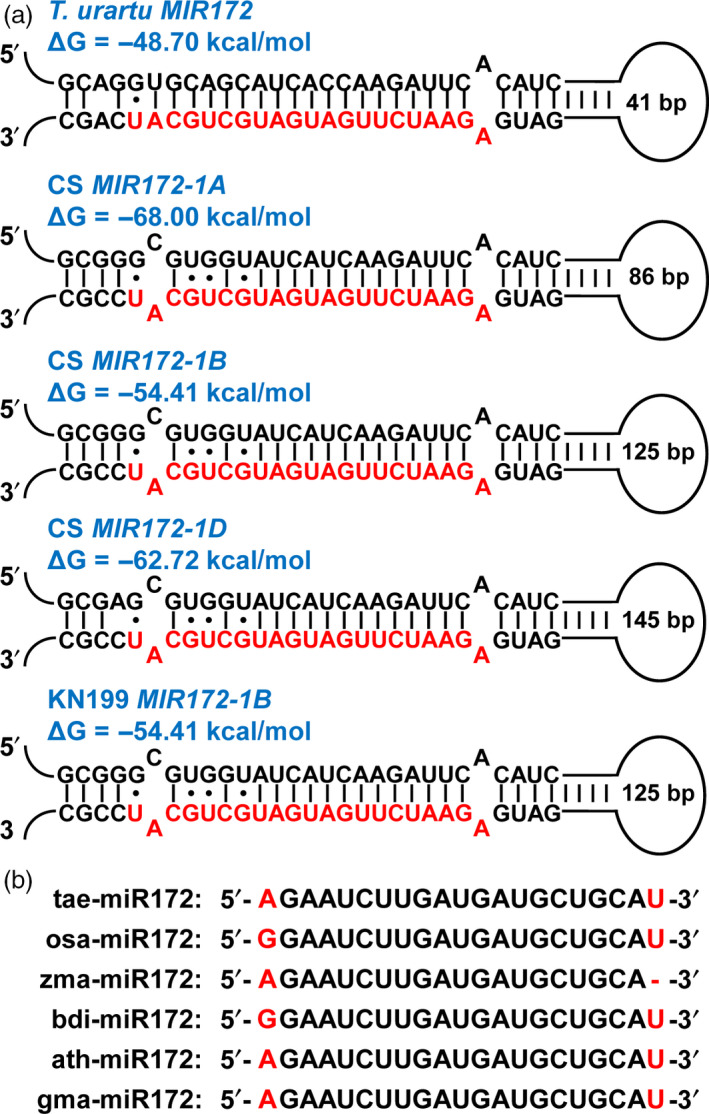
Identification of the *tae‐MIR172* precursors in bread wheat. (a) The secondary structures of *tae‐MIR172* precursors from the wheat A‐genome progenitor *Triticum urartu*, the bread wheat (*T. aestivum*) cultivar Chinese Spring (CS, chromosomes 1A, 1B and 1D) and cultivar Kenong199 (KN199, chromosome 1B). The mature tae‐miR172 sequences are highlighted in red. ΔG describes the minimum free energy (mfe) of the RNA structure. (b) Sequences of mature miR172 from different plant species. The single nucleotide variants were highlighted in red. tae, *T. aestivum*; osa, *Oryza sativa*; zma, *Zea mays*; bdi, *Brachypodium distachyon*; ath, *Arabidopsis thaliana*; gma, *Glycine max*.

Recent whole‐genome shotgun draft sequencing of the bread wheat A‐genome progenitor *Triticum urartu* revealed a group of scaffolds containing putative microRNA precursors (Ling *et al*., 2013). Among these scaffolds, scaffold7722 was predicted to contain a putative *MIR172* precursor (Ling *et al*., 2013). In this study, we further verified this *MIR172* precursor candidate by predicting its RNA secondary structure using the RNA‐folding program Mfold (Zuker, 2003). Certainly, a stable stem‐loop structure was obtained with low free energy (Figure 1a). More importantly, the miR172 mature sequence was contained in this structure within the 3′ arm of the hairpin (Figure 1a). These analyses suggest that the identified *MIR172* precursor meets the biogenesis criteria as a miRNA gene locus (Ambros *et al*., 2003; Meyers *et al*., 2008). Based on the information of scaffold7722, we screened the database of hexaploid wheat survey sequences (http://wheat-urgi.versailles.inra.fr/) (Deng *et al*., 2007; International Wheat Genome Sequencing Consortium, 2014) and found three sequences separately derived from the 1A, 1B and 1D chromosomes of the hexaploid bread wheat cultivar Chinese Spring (CS) (Figure S1). Based on these sequences, we designed specific primers and finally obtained a *MIR172* precursor derived from the 1B chromosome of the bread wheat cultivar Kenong199 (KN199) (Figure S1). Further analyses showed that all the identified *MIR172* precursors from the hexaploid bread wheat could form stable hairpin stem‐loop structures, resembling the *MIR172* precursor from bread wheat A‐genome progenitor *T. urartu* (Figure 1a). Meanwhile, the identified tae‐miR172 mature sequence is identical to the miR172 sequences from different plant species (Figure 1b), suggesting that the miR172 sequence is evolutionarily conserved.

### Over accumulation of tae‐miR172 causes the speltoid spike phenotype in bread wheat

**Figure 2:**
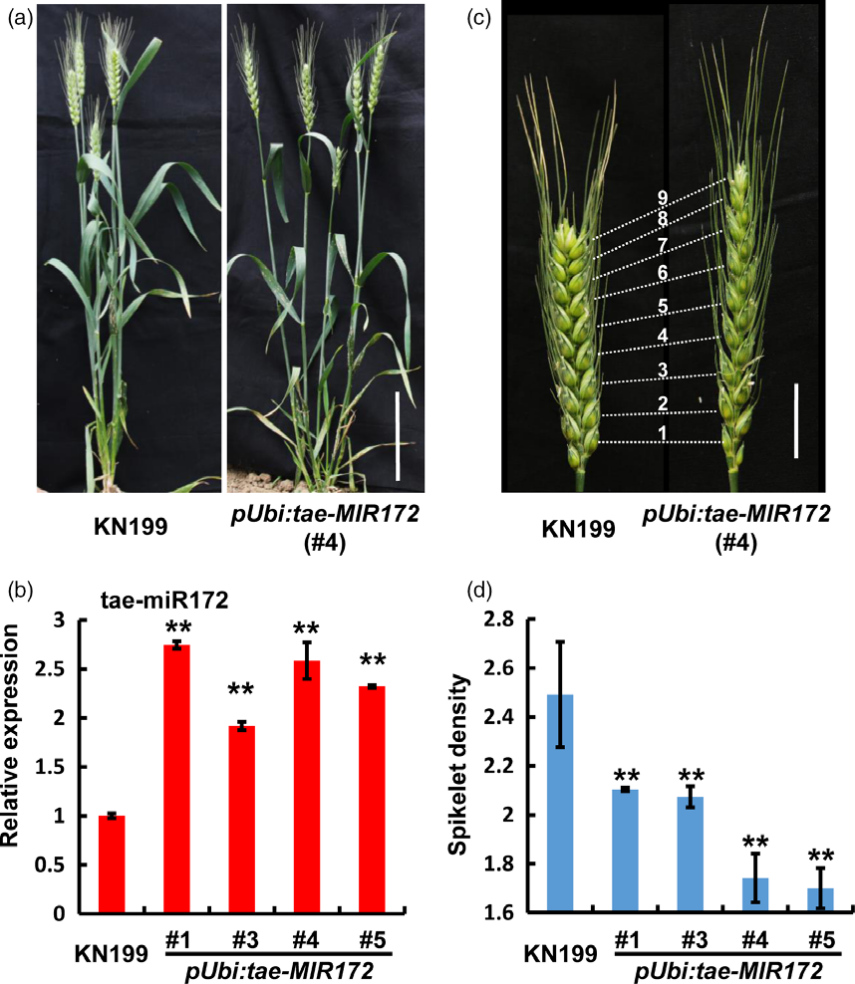
Over accumulation of tae‐miR172 leads to the speltoid spike phenotype in bread wheat. (a) Whole plant view of wild‐type (WT) KN199 (left) and *pUbi:tae‐MIR172* transgenic plants (right) at grain filling stage. Bar = 10 cm. (b) Quantification of tae‐miR172 in WT KN199 and *pUbi:tae‐MIR172* transgenic plants by stem‐loop quantitative reverse transcription PCR (qRT‐PCR) at the heading stage. The accumulation levels of tae‐miR172 were normalized against *TaU6*. Error bars represent standard deviations (SDs) among three independent replicates. (c) Spike morphology of KN199 (left) and *pUbi:tae‐MIR172* (right) at grain filling stage. Bar = 2 cm. (d) Spikelet densities (the number of spikelets per centimetre of spike length) of WT KN199 and *pUbi:tae‐MIR172* transgenic plants at the mature stage. Error bars denote SDs (*n* = 5). #1, #3, #4 and #5 represent independent transgenic lines; **P <* *0.01 (Student's *t‐*test).

To define the biological function of tae‐miR172 in bread wheat, we generated the transgenic bread wheat plants in KN199 background, in which the KN199 *MIR172‐1B* precursor was overexpressed driven by the ubiquitin promoter. As shown in Figures 2a and S2, the whole plant morphology of *pUbi:tae‐MIR172* transgenic bread wheat plants, including plant height and tillering, is largely similar to those of wild‐type (WT) KN199, even though more than two fold up‐regulation of miR172 was detected in independent *pUbi:tae‐MIR172* transgenic lines (Figure 2b). Nevertheless, the spike architecture of the *pUbi:tae‐MIR172* transgenic plants was significantly altered, displaying the speltoid spike phenotype, that is a spear‐shaped spike with elongated rachis (Figures 2c and S2). To quantitatively evaluate the spike phenotype, we determined the spikelet densities (the number of spikelets per centimetre of spike length) and revealed that the spikelet densities from different *pUbi:tae‐MIR172* transgenic lines were all significantly reduced compared with that in WT KN199 (Figure 2d). Together, these observations suggest that miR172 may be specifically involved in the regulation of bread wheat spike architecture.

### The domestication gene *Q* is targeted by tae‐miR172

To better understand the molecular basis for the tae‐miR172 regulation on spike architecture, we focused on the identification of potential target genes of tae‐miR172 in bread wheat. In this study, we predicted the targets of tae‐miR172 using the web‐based plant small RNA target analysis tool *psRNATarget* (Dai and Zhao, 2011) by setting the maximum expectation as 3.0 and maximum energy to unpair the target site (UPE) as 25.0. Finally, 22 nucleotide sequences were identified as the candidates of tae‐miR172‐targeting genes (Table S1).

**Figure 3:**
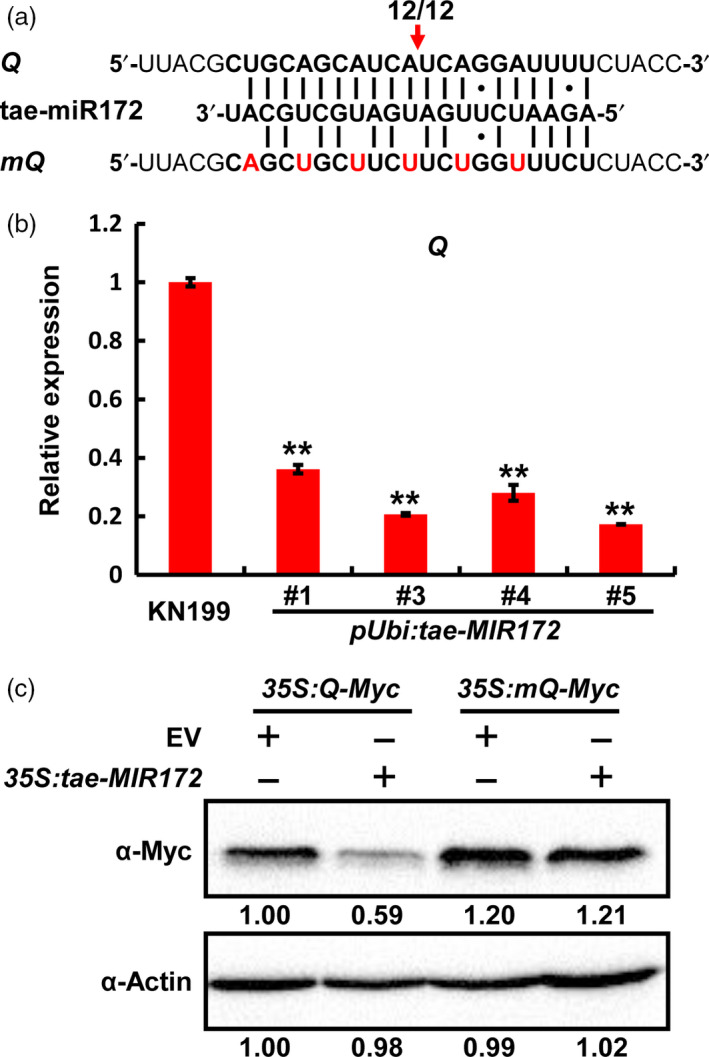
*Q* is a target gene of tae‐miR172 in bread wheat. (a) Identification of cleavage site in *Q* by 5′ RACE. The red arrow indicates the cleavage site, and numbers below the arrow show the frequency of clones with matching 5′RACE products from this site out of total clones identified by sequencing. *mQ*, tae‐miR172‐cleavage resistant form of *Q*. (b) Determination of *Q* transcript levels by qRT‐PCR in WT KN199 and *pUbi:tae‐MIR172* lines at heading stage. The flag leaves were collected for the determination, and the transcript levels of *Q* were normalized against the internal control gene *TaGAPDH*. Error bars represent SDs among three independent replicates. **P < 0.01 (Student's *t‐*test). (c) Transient expression assay in *N. benthamiana* confirming that *Q* is a target gene of tae‐miR172. *35S:Q‐Myc* and *35S:mQ‐Myc* were separately co‐expressed with *35S:tae‐MIR172* in *N. benthamiana* leaves, and the protein levels of Q‐Myc and actin were determined by Western blotting using α‐Myc or α‐Actin antibodies. Numbers below the blots represent relative protein levels as calculated by Image J software. In each experiment, four independent leaves were analysed, and three replicates were performed with similar results.

Among these tae‐miR172 targets, a certain number of sequences are described to encode the AP2‐containing transcription factor Q in bread wheat (Table S1). To further experimentally confirm whether *Q* is the target of miR172, we first determined whether tae‐miR172 could trigger cleavage on *Q* transcripts *in vivo* using the previously described rapid amplification of cDNA 5′ ends (5′ RACE) method (Liu *et al*., 2014). Our results showed that *Q* mRNA could indeed be cleaved *in vivo* at the specific cleavage site at the position 10–11 of tae‐miR172 from its 5′ end (Figures 3a and S3), which was previously identified as a canonical miRNA cleavage site (Peters and Meister, 2007). To further validate the cleavage of tae‐miR172 on *Q* mRNAs, we determined the *Q* transcript levels in WT KN199 as well as *pUbi:tae‐MIR172* transgenic plants and found that *Q* was significantly down‐regulated upon the overexpression of tae‐miR172 in *pUbi:tae‐MIR172* plants, compared with WT KN199 (Figure 3b). We next determined the suppression effect of tae‐miR172 on *Q* by transient expression assays in *Nicotiana benthamiana*. As expected, co‐expression of *tae‐MIR172* significantly decreased the accumulation of Q protein (Figure 3c, lane 2), compared with the empty vector (EV) control (Figure 3c, lane 1). By contrast, the alteration of nucleotides at the miR172‐targeting site in *Q* (*mQ* shown in Figure 3a) completely blocked the suppression effect of miR172 on Q protein accumulation (Figure 3c, lanes 3 and 4), suggesting that the suppression of tae‐miR172 on *Q* is largely dependent on the strict reverse complementation between miR172 and *Q* transcripts.

As the wheat *Q* gene has been reported to influence a number of domestication‐related traits such as the rachis fragility and threshability (Simons *et al*., 2006; Zhang *et al*., 2011), we tested whether these domestication‐related traits were affected in our *pUbi:tae‐MIR172* transgenic plants. Indeed, not like the loosely held seeds in WT KN199, we observed a non‐free‐threshing phenotype in all the tested *pUbi:tae‐MIR172* transgenic plants when grown in the field conditions (Figure S4). More importantly, the spikes from the *pUbi:tae‐MIR172* transgenic plants were more easier to break into individual spikelets due to more fragile rachis (Figure S4).

In summary, our data strongly suggest that tae‐miR172 targets *Q* gene, and directs the cleavage of *Q* transcripts in bread wheat.

### The Q protein acts as a nuclear transcriptional repressor

**Figure 4:**
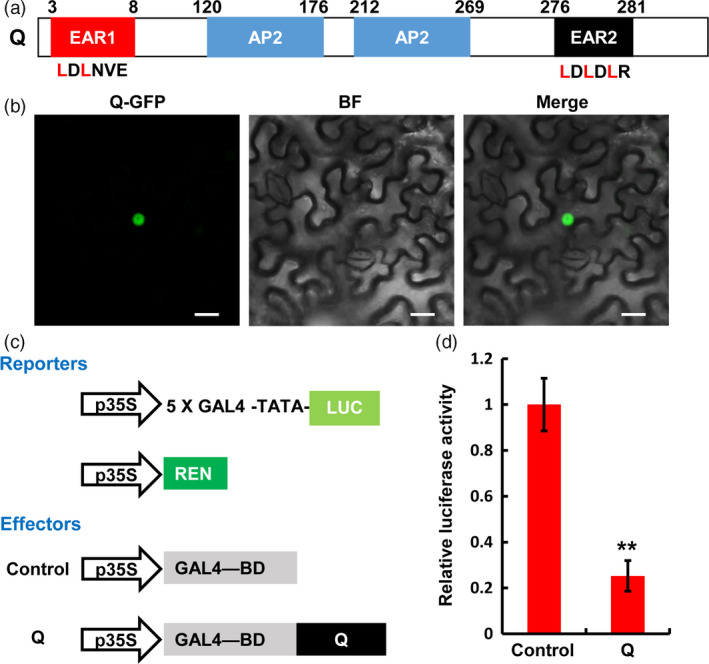
*Q* encodes an AP2 family transcription factor with transcription repression activity. (a) Schematic diagram of the domain structure of Q protein. The EAR (ethylene‐responsive element binding factor‐associated amphiphilic repression) motifs located at the N‐ or C‐terminus of Q were annotated as EAR1 (LDLNVE) and EAR2 (LDLDLR), respectively. (b) Subcellular localization of Q. The *35S:Q‐GFP* was expressed in *N. benthamiana* leaves by *Agrobacterium*‐mediated infiltration. GFP signal was detected 48 h post infiltration (hpi). In each experiment, four leaves were analysed. Three replicates were performed independently with similar results. BF, bright field. Scale bars, 20 μm. (c) and (d) Transient expression assay in *N. benthamiana* protoplasts illustrating the transcriptional repression activity of Q. The reporters and effectors used in the assay were generated as shown in (c). The activities of firefly luciferase (LUC) and renilla luciferase (REN) were determined 16 h post‐transformation. The relative luciferase activities in control and *Q*‐expressed samples as shown in (d) were calculated by normalizing the LUC values against REN. Error bars indicate SDs among three independent replicates. **P < 0.01 (Student's *t‐*test).

The *Q* gene encodes a member of the AP2 family transcription factors, which contains an atypical EAR motif (LDLNVE) on the N‐terminal region, two AP2 domains in the middle region and a typical EAR motif (LDLDLR) on its C‐terminus (Figure 4a). To determine the subcellular localization of Q protein, we fused green fluorescent protein (GFP) with Q for the transient expression assay in *N. benthamiana*. As shown in Figure 4b, the fluorescence signal of GFP could be observed exclusively in the nuclei of *N. benthamiana* epidermal cells, confirming that Q functions as a nucleus‐localized transcriptional regulator. Further, we determined the transcriptional activity of Q in a transient dual‐luciferase expression system using *N. benthamiana* protoplasts. In this assay, the firefly luciferase (*LUC*) gene was fused to a 5 × GAL4 binding site to generate the reporter, and the renilla luciferase (*REN*) gene driven by *35S* promoter was used as the internal control (Figure 4c). Meanwhile, the effector plasmid was constructed by fusing the *Q* coding sequence to the GAL4 DNA binding domain (GAL4‐BD) (Figure 4c). Bioluminescence determination revealed that the expression of Q led to an obvious down‐regulation of the relative luciferase activity, compared to the EV control (Figure 4d). These results indicate that Q potentially acts as a transcriptional repressor in plant cell nuclei.

### Molecular characterization of the transcriptional co‐repressor TaTPL in bread wheat

**Figure 5:**
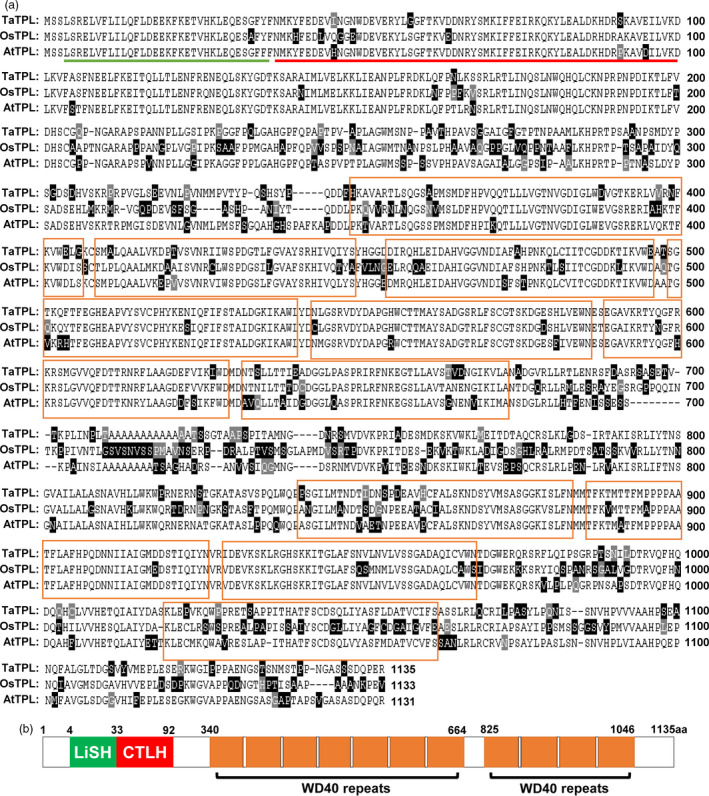
Identification of TaTPL in bread wheat. (a) Sequence alignment of TaTPL, OsTPL (LOC_Os08g0162100) and AtTPL (At1g15750) proteins. The graphic view of alignment was generated by MegAlign using Clustal W method. The black or grey shade represents the similarity. The green line represents LiSH (lissencephaly type‐1‐like homology) domain, while the red line denotes CTLH (C‐terminal to LiSH) domain. The WD40 repeats are marked by orange boxes. (b) Schematic diagram of the conserved domains in TaTPL.

Our sequence analysis revealed that the Q protein contains two EAR motifs (Figure 4a). Thus, we hypothesized that Q might physically interact with the transcriptional co‐repressors of TPL/TRPs. To this end, we identified the *TaTPL* genes from bread wheat cultivar KN199. First, we used the coding sequence (CDS) of *OsTPL* (LOC_Os08g0162100) as a query to search the wheat survey sequences using BLAST (http://wheat-urgi.versailles.inra.fr/) (Deng *et al*., 2007; International Wheat Genome Sequencing Consortium, 2014) and identified one putative *TPL*‐like gene that shares high‐level identity with *OsTPL*. Based on this sequence, we next designed gene‐specific primers and cloned its homologous genes from bread wheat cultivar KN199. Totally, two highly conserved *TPL*‐like sequences with single nucleotide polymorphisms (SNPs) were obtained from KN199 (Figures S5 and S6). By sequence comparison, the two *TPL*‐like sequences were separately located on chromosomes 4A and 4D, which show ~71% and ~72% similarities with *OsTPL* and *AtTPL*, respectively. Thus, we named these two coding sequences as *TaTPL‐4A* and *TaTPL‐4D*. Further analysis revealed that the protein sequences encoded by *TaTPL‐4A* and *TaTPL‐4D* are completely identical and share ~67% and ~72% identity with OsTPL and AtTPL at the protein level (Figure 5a), suggesting that TPL proteins are highly conserved in different plant species.

More significantly, similar to the TPL family proteins in *Arabidopsis* and rice (Ke *et al*., 2015; Szemenyei *et al*., 2008), TaTPL also contains the conserved LiSH and CTLH motifs in its N‐terminus and WD40 repeats in the C‐terminal region (Figure 5b), indicating the conserved molecular structure of TaTPL as the putative transcriptional co‐repressor in bread wheat.

### Transcriptional expression analyses of *Q* and *TaTPL*

**Figure 6:**
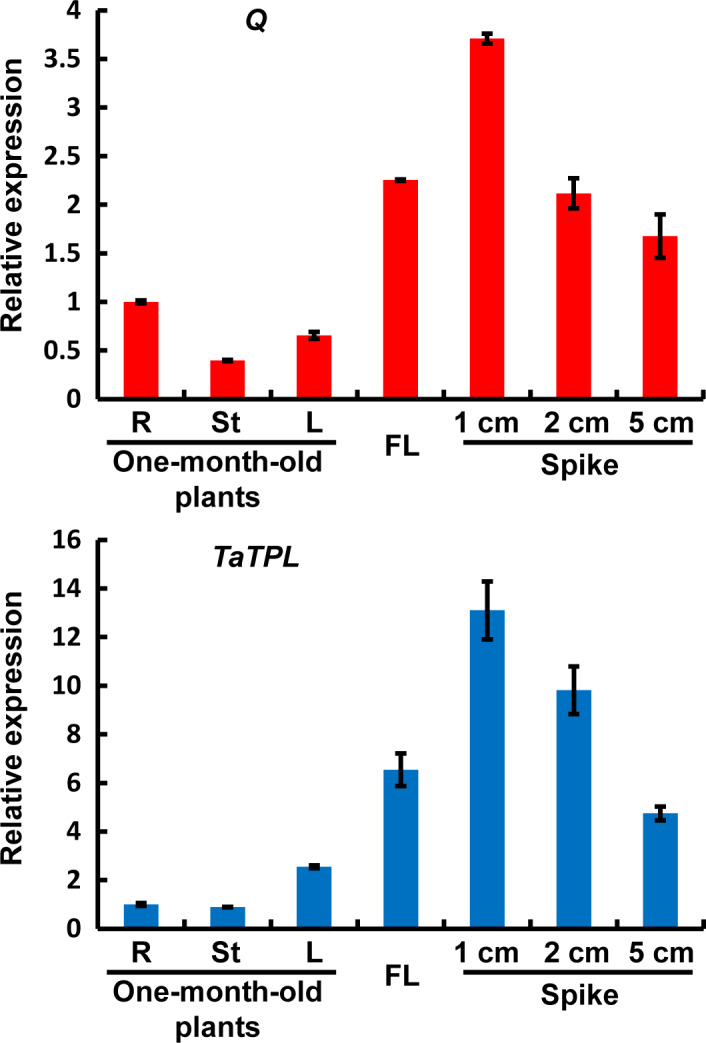
Determination of the transcript levels of *Q* and *TaTPL* in different tissues/stages in bread wheat KN199. R, root tip; St, stem; L, leaf; FL, flag leaf. The levels of *Q* and *TaTPL* transcripts were normalized against *TaGAPDH*. Error bars represent SDs among three independent replicates.

To investigate the biological correlation between *Q* and *TaTPL*, we examined their expression patterns in different tissues/stages in bread wheat. For this experiment, the root tips (R), the stem (St) and the fully expanded leaf (L) from 1‐month‐old bread wheat plants, as well as the flag leaf (FL) from adult plants, and young spikes at different developmental stages (1, 2 and 5 cm in length) were individually collected. Interestingly, our quantitative real‐time PCR analyses revealed that the transcriptional expression patterns of *Q* and *TaTPL* were quite similar (Figure 6). Although the two genes were constitutively expressed in all tested tissues, their transcripts were predominantly accumulated in flag leaves and spikes, with highest levels in 1‐cm spikes (Figure 6). In addition, we noticed that the transcript levels of *Q* and *TaTPL* in spikes gradually decreased during the development of spike (Figure 6), indicating that *Q* and *TaTPL* might be involved in the early‐stage development of bread wheat spike.

### Q physically interacts with the co‐repressor TaTPL

**Figure 7:**
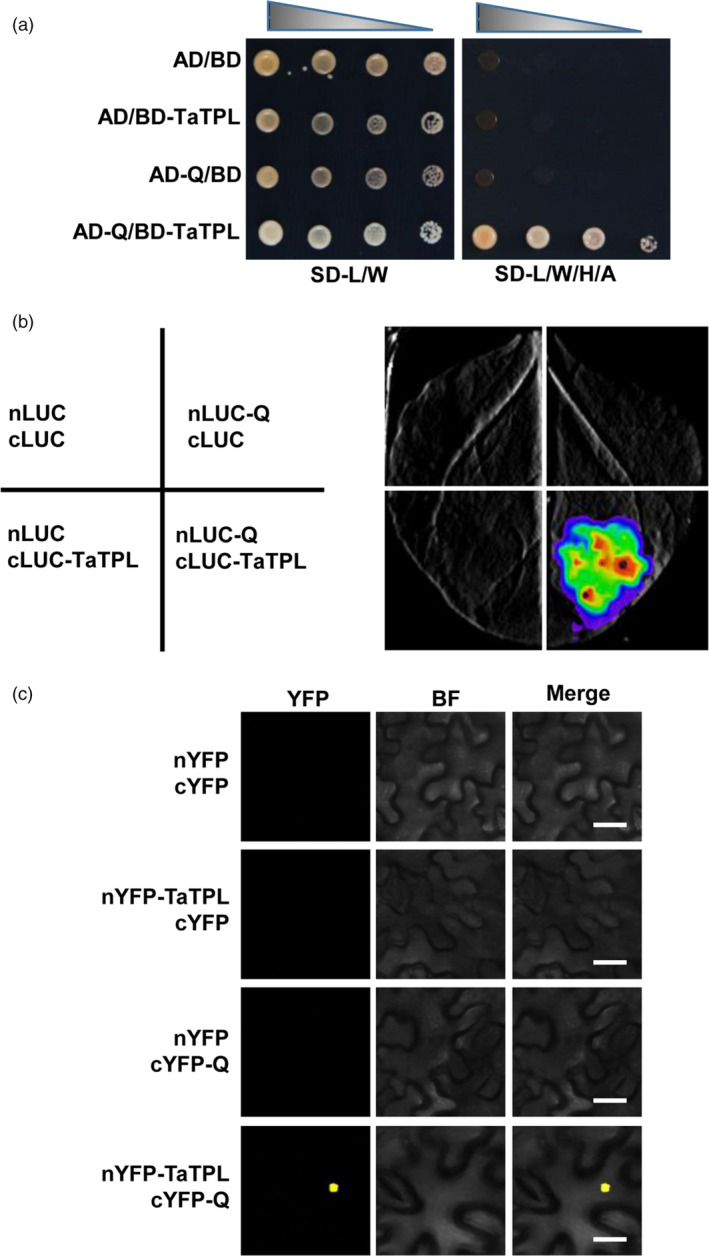
Q directly interacts with TaTPL. (a) Yeast two‐hybrid (Y2H) assay showing the interaction between Q and TaTPL. SD‐L/W, synthetic dextrose medium lacking Leu and Trp; SD‐L/W/H/A, synthetic dextrose medium lacking Leu, Trp, His and Ade. (b) Luciferase (LUC) complementation imaging (LCI) assay illustrating that Q could interact with TaTPL in *N. benthamiana*. Luciferase signals were detected 48 hpi. (c) Bimolecular fluorescence complementation (BiFC) assay confirming the physical interaction of Q with TaTPL in *N. benthamiana*. YFP fluorescence was detected 48 hpi. BF, bright field. Scale bars, 20 μm. In each experiment in (b) and (c), six leaves were employed for the analyses. All the above experiments were independently repeated for three times with similar results.

Further, we determined whether Q protein could physically interact with the identified transcriptional co‐repressor TaTPL. First, we performed yeast two‐hybrid (Y2H) assays. Considering that the *TaTPL‐4A* and *TaTPL‐4D* genes encode the same protein sequence, here we used *TaTPL‐4D* for the assays. As shown in Figure 7a, an obvious interaction was indeed observed between Q and TaTPL in the AH109 yeast (*Saccharomyces cerevisiae*) cells. To further confirm this interaction *in planta*, we carried out firefly luciferase (LUC) complementation imaging (LCI) assay in *N. benthamiana* (Song *et al*., 2011). Results showed that strong LUC activity was exclusively observed in nLUC‐Q and cLUC‐TaTPL co‐expressed samples, but not in the negative controls (Figure 7b), indicating that Q and TaTPL could physically associate with each other *in vivo*. Moreover, parallel biomolecular fluorescence complementation (BiFC) assays in *N. benthamiana* also confirmed that Q could directly interact with TaTPL in the nuclei of plant cells (Figure 7c).

Together, these data strongly suggest that Q physically associate with the transcriptional co‐repressor TaTPL in the nuclei, which is consistent with the above finding that Q functions as a functional transcription factor with transcriptional repression activity (Figure 4d).

### The N‐terminal EAR motif of Q protein mediates the interaction with TaTPL

**Figure 8:**
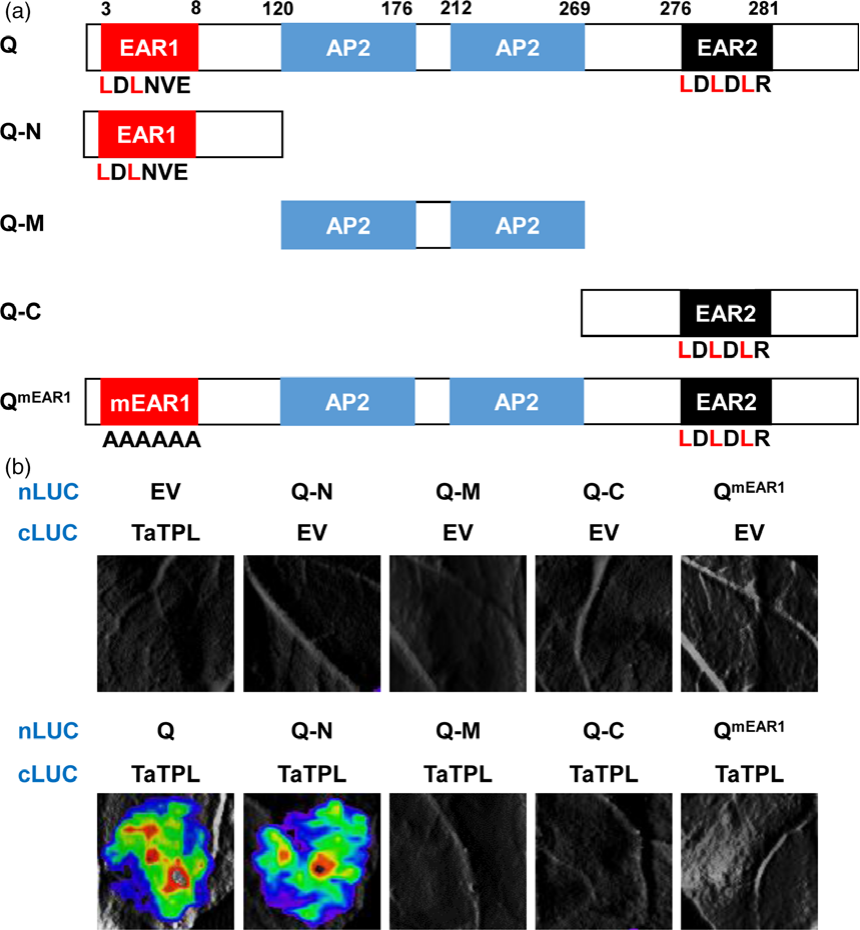
The N‐terminal EAR motif of Q mediates its interaction with TaTPL. (a) The truncated or mutated versions of Q employed in the interaction assays in *N. benthamiana*. Q‐N, 1–199 amino acids (aa); Q‐M, 120–275 aa; Q‐C, 276–447 aa; Q^mEAR1^, the full‐length Q with mutation in EAR1 motif. (b) LCI assays mapping the interaction domain of Q with the full‐length TaTPL. The interaction signals were detected at 48 hpi. In each experiment, six *N. benthamiana* leaves were infiltrated for analysis, and similar results were observed. Three independent replicates were performed. EV, empty vector.

As described above, Q contains two different EAR motifs (Figure 4a). To further define which EAR motif is essentially required for the interaction with TaTPL, we generated truncated forms of Q. As shown in Figure 8a, Q‐N and Q‐C separately represent the N (1–199 amino acids)‐ and C (276–447 aa)‐terminal domains of Q, which contain the EAR1 and EAR2 motifs, respectively; while Q‐M denotes Q middle domain (120–275 aa) containing the two conserved AP2 domains. The LCI assays revealed that similar to the full‐length Q protein, Q‐N, but not Q‐M or Q‐C, reserved the interaction signal (Figure 8b), indicating that the EAR1 motif on the N‐terminus of Q might be essential for the physical interaction with TaTPL. To further confirm this conclusion, we generated a mutated Q form (Q^mEAR1^), in which the EAR1 motif (LDLNVE) was substituted by the tandem Alanine (AAAAAA). As expected, Q^mEAR1^ failed to interact with TaTPL, illustrating that the N‐terminal EAR1 motif of Q is required for its interaction with TaTPL.

### The N‐terminal CTLH motif of TaTPL mediates the interaction with Q protein

**Figure 9:**
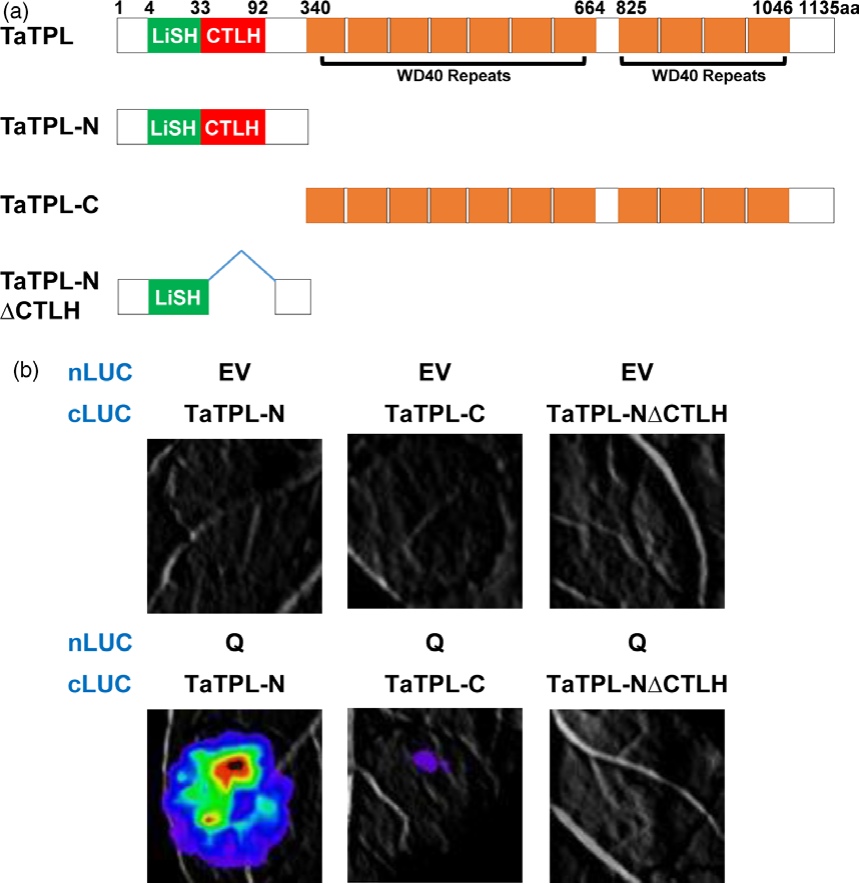
The CTHL motif of TaTPL is required for the physical association with Q protein. (a) The truncated versions of TaTPL used in the interaction assays. TaTPL‐N, 1–339 aa; TaTPL‐C, 340–1135 aa; TaTPL‐N∆CTHL, the TaTPL‐N domain without the CTHL motif (34–92 aa). (b) LCI assays illustrating the interaction of truncated versions of TaTPL with the full‐length Q protein in *N. benthamiana*. Signals were collected at 48 hpi. Six *N. benthamiana* leaves were analysed in each experiment, and three independent replicates were performed with similar results.

Based on the highly conserved domains contained in TaTPL, we generated TaTPL derivatives, including TaTPL‐N (1–339 aa) and TaTPL‐C (340–1135 aa), to map the domain responsible for its interaction with Q (Figure 9a). Interestingly, a strong interaction signal was only observed between Q and TaTPL‐N (Figure 9b). Further, we deleted the conserved CTLH motif to create a TaTPL‐N∆CTLH fragment. Results showed that the deletion of CTLH motif in TaTPL abolished its interaction with Q (Figure 9b). Together, these results suggest that the CTLH motif of TaTPL is essentially needed for the physical interaction with Q protein.

### The TaTPL‐interacting EAR motif of Q is required for its transcriptional repression activity

**Figure 10:**
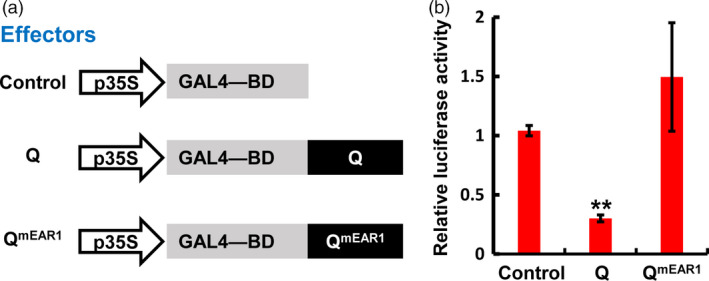
Transient expression assay in *N. benthamiana* protoplasts illustrating the transcriptional repression activity of wild‐type and mutated Q. (a) The effectors used in the assay. (b) Relative luciferase activities in effector‐expressed samples. The activities of LUC and REN were determined 16 h post‐transformation, and the relative luciferase activities in different samples were calculated by normalizing the LUC values against REN. Error bars indicate SDs among three independent replicates. **P < 0.01 (Student's *t*‐test).

Based on our findings that Q function as a transcriptional repressor and physically interacts with the transcriptional co‐repressor TaTPL through its N‐terminal EAR motif (Figures 4 and 8), we speculated that the N‐terminal EAR motif of Q might be responsible for its transcriptional repression activity through recruiting the transcriptional co‐repressor TaTPL. To test this hypothesis, we determined the transcriptional activity of Q^mEAR1^, in which the N‐terminal EAR motif was mutated as shown in Figure 8a, in a transient dual‐luciferase expression system using *N. benthamiana* protoplasts. Here we fused the *Q* ^*mEAR1*^ coding sequence to the GAL4‐BD to generate the Q^mEAR1^ effector (Figure 10a), and co‐expressed with the reporter *LUC* and the internal control *REN* as shown in Figure 4c. Consistent with our above results (Figure 4d), the expression of Q effector led to a repression of the relative luciferase activity (Figure 10b). However, not like Q, the Q^mEAR1^ effector did not show any repressive activity when compared with the control (Figure 10b), suggesting that the transcriptional repression activity of Q was abolished when the N‐terminal EAR motif was mutated. These results indicate that the N‐terminal EAR motif of Q is essentially required for its transcriptional repression activity and the interaction with the transcriptional co‐repressor TaTPL.

## Discussion

*Q* is a critical domestication gene in polyploid wheat that is responsible for the widespread cultivation of the bread wheat (Faris and Gill, 2002; Muramatsu, 1963; Simons *et al*., 2006; Zhang *et al*., 2011). Studies reveal that *Q* affects a series of domestication characters in wheat, including the seed free‐threshing and rachis fragility (Zhang *et al*., 2011). More significantly, *Q* is involved in the control of spike morphology, considering that the *q* allele in wild wheat causes a speltoid spike phenotype that displays elongated rachis and spear‐shaped spike morphology. In this study, we reproduced the speltoid spike phenotype by overexpression of tae‐miR172 in bread wheat plants KN199 (Figures 2c and S2), suggesting that tae‐miR172 should be involved in same signalling pathway with *Q* in the regulation of bread wheat spike architecture.

Several lines of evidence indeed show that the domestication gene *Q* is targeted and regulated by tae‐miR172. First, the mature tae‐miR172 sequence is highly complemental to the target region of *Q* mRNA (Figure 3a). Second, the *Q* mRNA levels are markedly reduced in the tae‐miR172 overexpression bread wheat plants (Figure 3b). Third, 5′ RACE assays showed that the *Q* mRNAs are cleaved in a specific site by tae‐miR172 (Figure 3b). Fourth, the Q accumulation could also be significantly suppressed by the expression of tae‐miR172 in the transient expression system (Figure 3c). Taken together, our present data support the hypothesis that tae‐miR172 may regulate wheat spike architecture, especially the spikelet density mainly through the repression of *Q*. Interestingly, during the review of our study, a study reported that the nucleotide mutations in the miR172‐targeting site of *Q*, which interfere with the miR172‐mediated suppression and thus lead to *Q* accumulation, resulted in increased spikelet densities in bread wheat (Xu *et al*., 2017). This report, from another point of view, well supports our conclusion that the miR172‐*Q* module is crucial in the regulation of wheat spike architecture.

Besides the *Q* gene, several other genes encoding AP2‐containing transcription factors were also predicted to be potential targets of tae‐miR172 (Table S1). Generally, these AP2‐like transcription factors may function redundantly *in planta* to influence multiple signalling pathways. So far, we could not rule out the possibility that besides Q, other AP2‐like transcription factors might also participate in the modulation of bread wheat spike architecture. Thus, it would be intriguing to test the biological functions of these transcription factors in bread wheat, and also their genetic relationship with the miR172‐*Q* module.

Transcriptional degradation and translational inhibition are two major mechanisms for miRNAs to direct the regulation of their target genes. Previous study in *Arabidopsis thaliana* revealed that miR172 likely suppresses its target gene *APETALA2* mainly through translational suppression, as the *AP2* mRNA abundance was not affected by the overexpression of miR172 (Chen, 2004). However, in this study, we clearly observed the miR172‐guided cleavage of *Q* transcripts by 5′ RACE in our assays (Figure 3a), which was also supported by very recent studies (Debernardi *et al*., 2017; Greenwood *et al*., 2017; Xu *et al*., 2017). More importantly, the *Q* mRNA levels were dramatically decreased in tae‐miR172 overexpression bread wheat plants, compared with those in the WT control (Figure 3b), suggesting that the transcriptional degradation is the major mechanism employed by tae‐miR172 in suppression of *Q* in bread wheat. In consistent with our findings, some works in *Arabidopsis* and rice also detected the cleavage event of *AP2* mRNA directed by miR172 (Kasschau *et al*., 2003; Varkonyi‐Gasic *et al*., 2012; Zhu *et al*., 2009). These reports, together with our results, promote us to propose that miR172 can regulate its target genes through both mechanisms of transcript cleavage and translational inhibition, but chose one as the major mode of action, depending on the specific conditions and plant species.

Although it has been shown that *Q* gene plays important roles in the domestication of bread wheat (Debernardi *et al*., 2017; Greenwood *et al*., 2017; Simons *et al*., 2006; Xu *et al*., 2017; Zhang *et al*., 2011), the underlying mechanisms of Q protein action remain unclear. In this study, we showed that Q protein is a transcription repressor with two EAR motifs (Figure 4). Previous study in *Arabidopsis* has reported that AP2 transcription factor functions as the transcriptional repressor through the recruitment of the co‐repressor TOPLESS (Krogan *et al*., 2012). Thus, we asked whether the transcriptional repressor Q could also physically associate with TaTPL. Using LCI, BiFC and Y2H analyses, we confidently demonstrated that Q could indeed interact with the transcriptional co‐repressor TaTPL (Figure 7). Interestingly, our data showed that the N‐terminal atypical EAR motif (LDLNVE), but not the C‐terminal typical EAR motif (LDLDLR) of Q protein is essentially required for the interaction with the conserved CTLH motif of TaTPL. More importantly, our assays confirmed that the N‐terminal EAR motif of Q is essentially required for the Q‐mediated transcriptional repression activity (Figure 10). These results further support the hypothesis that TPL is indispensable for the repression activity of Q. Previous studies indeed showed that TPL‐like proteins are involved in the appropriate establishment of spike/panicle architecture (Gallavotti *et al*., 2010; Yoshida *et al*., 2012). In maize, REL2 was identified as a TPL‐like transcriptional co‐repressor and could physically interact with RA1 to repress the downstream genes that are involved in the spikelet‐pair meristem determinacy pathway (Gallavotti *et al*., 2010). In support of this, the *rel2* mutant plants showed enhanced inflorescence primary branching phenotype (Gallavotti *et al*., 2010). Our data, together with the above reports, lead us to propose a molecular mechanism in regulating bread wheat spike architecture: the miR172‐regulated Q, functions as a transcription factor with transcriptional repression activity, should negatively regulate the expression of numerous downstream target genes through the recruitment of TOPLESS co‐repressors, and subsequently suppresses the speltoid phenotype in bread wheat. Nevertheless, to further experimentally confirm the potential relationship between Q and TaTPL in spike architecture determination, more works are eagerly needed to identify the downstream genes that are directly regulated by Q, which will further deepen our knowledge with respect to the bread wheat spike architecture regulatory network.

## Experimental procedures

### Gene transformation, plant materials and growth conditions

In this study, the bread wheat (*T. aestivum*) cultivar Kenong199 (KN199), which is one of the major bread wheat cultivars in China and also easy for gene transformation, was employed as the receptor material to generate the transgenic plants. The 1‐month‐old embryogenic calli of KN199 were selected as the receptor materials, and the gene transformation was performed using a PDS1000/He particle bombardment system (Bio‐Rad, Hercules, CA) with a distance of 6.0 cm from the stopping plate at helium pressure 1100 psi, as described previously (Shan *et al*., 2013).

For observation of bread wheat spike phenotypes, seeds of the wild‐type (WT) KN199 and transgenic bread wheat were first germinated at 22 °C, and transferred into 4 °C cold room for a 1‐month vernalization. Then, the seedlings were transplanted in the experimental field under natural conditions. The spike phenotypes were collected at grain filling and mature stages, and the spikelet densities (the number of spikelets per cm of spike length) were determined at the mature stage.

*Nicotiana benthamiana* was grown in glasshouse at 22 °C with a 16‐h light/8‐h dark cycle.

### Prediction of miRNA targets and *MIRNA* secondary structure

In this study, the web‐based plant small RNA target analysis tool *psRNATarget* was used for the prediction of potential target genes of tae‐miR172 in bread wheat (Dai and Zhao, 2011). The wheat unigene library (DFCI Gene Index, version 12, released on 2010.04.18) was employed as the database, and the mature tae‐miR172 was used as the query. Maximum expectation was set as 3.0, length for complementarity scoring 20 and maximum energy to un‐pair the target site (UPE) as 25.0.

The secondary structure of *MIRNA* was predicted using the RNA‐folding program Mfold (Zuker, 2003), and the minimum free energy (mfe) of the RNA structure, described as ΔG, was calculated simultaneously.

### RNA extraction and gene expression analysis

Total RNA was extracted using Trizol (Invitrogen) reagent. For microRNA reverse transcription, the stem‐loop primer was designed according to the sequence of mature miR172 (Chen *et al*., 2005). About 2 μg of total RNA and Moloney murine leukaemia virus reverse transcriptase (M‐MLV, Invitrogen) were used for the reverse transcription. The *TaU6* gene was reversely transcribed simultaneously as the internal control gene. cDNA was obtained using 2 μg total RNA and All‐In‐One RT MasterMix (Applied Biological Materials) following the manufacturer's instruction. For real‐time quantification, SYBR^®^ Premix Ex Taq™ (Perfect Real Time, TaKaRa) was used. The expression levels of coding genes and miRNAs were separately normalized to *TaGAPDH* and *TaU6*.

### Validation of miRNA cleavage by 5′ RACE assay

The 5′ RACE assay was performed using the RLM‐RACE kit (TaKaRa, Code D315), following the procedure reported before (Liu *et al*., 2014). Briefly, about 2 μg of total RNAs of KN199 was used to directly ligate the RNA oligo adaptor, followed by reverse transcription using random primer. The 5′ RACE outer primer together with gene‐specific outer primer and the 5′ RACE inner primer coupled with gene‐specific inner primer were separately used for the first and second round nested PCR. The PCR product was extracted by gel purification and ligated to cloning vector (*pEASY*‐Blunt, Transgen biotech CB101) for sequencing.

### Confirmation of the miRNA target by transient expression assay

Transient expression assay in *N. benthamiana* was performed to confirm the miRNA target (Liu *et al*., 2014). Briefly, *Agrobacterium tumefaciens* suspension expressing the miRNA precursor was first infiltrated into *N. benthamiana* leaves; meanwhile, *A. tumefaciens* harbouring empty vector was infiltrated as the negative control. After 24 h, *A. tumefaciens* containing the constructs expressing the target genes of interest were infiltrated into the same position of *N. benthamiana* leaves. Samples were collected 36–48 h post infiltration (hpi). Total proteins were extracted using 2× Laemmli buffer (Laemmli, 1970) and detected by immunoblotting using anti‐Myc antibody (1 : 5000; Roche, 11667149001) and anti‐mouse IgG conjugated with horseradish peroxidase (HRP) (1 : 75 000; Sigma, A9044‐2ML). The levels of actin were detected by anti‐β‐actin antibody (1 : 5000; CWBIO, CW0264) and anti‐mouse IgG as the internal control.

### DNA constructs and primers

DNA constructs used in the study were generated based on construction methods following the classic molecular biology protocols and Gateway technology (Invitrogen). All details of DNA constructs used in this study were listed in Table S2. All the primers used for generation of these constructs were shown in Table S3.

### Luciferase transient transcriptional repression assay

Two kinds of reporters were used in the assay. One contains the firefly luciferase (*LUC*) gene fused with 5× GAL4 binding site, and the other is a plasmid expressing the renilla luciferase (*REN*) gene as the internal control. The effector plasmid (*GAL4‐BD‐Q*) was co‐transformed with the two reporters, and the activities of LUC and REN were separately determined 24 h post‐transformation using Dual‐Luciferase^®^ Reporter Assay System (Promega, E1910).

### Yeast two‐hybrid assay

For yeast two‐hybrid analysis, the GAL4‐AD and GAL4‐BD derivatives were generated by fusing the coding sequences of *TaTPL* and *Q* and co‐transformed into the yeast (*Saccharomyces cerevisiae*) strain AH109. The yeast cells were first selected on synthetic dextrose medium lacking Leu and Trp (SD‐L/W) and then transferred to the SD medium lacking Leu, Trp, His and adenine (SD‐L/W/H/A) for interaction analysis.

### LCI assay

The LCI assays were performed in *N. benthamiana* leaves (Sun *et al*., 2013). The full‐length or truncated versions of target proteins were separately fused with the N‐ and C‐terminal part of the firefly luciferase LUC, and expressed in *N. benthamiana* leaves by *A. tumefaciens*‐mediated infiltration. The LUC activity was analysed at 48 hpi.

### Subcellular localization analysis

The coding sequence of *Q* was cloned into the pGWB5 vector (Nakagawa *et al*., 2007) and then was transformed into *A. tumefaciens* GV3101. The *Agrobacterium* was infiltrated into *N. benthamiana* leaves, and the fluorescence signal of green fluorescent protein (YFP) was observed at 48 hpi.

### BiFC assay

BiFC was performed in *N. benthamiana* leaves to detect Q‐TaTPL interaction (Lu *et al*., 2010). Briefly, the full‐length coding sequences of *TaTPL* and *Q* were separately ligated into the pEarleygate201 and pEarleygate202 vectors, and co‐expressed in *N. benthamiana* leaves through *A. tumefaciens*‐mediated transient expression. The fluorescence signal of yellow fluorescent protein (YFP) was observed at 48 hpi.

## Conflict of interest

The authors declare no conflict of interests.

## Author contributions

JS designed research; PL, JL and HD performed the experiments; JS, PL and JL analysed the data and wrote the manuscript.

## Supporting information


**Figure S1** Sequence alignment of *tae‐MIR172* precursors from chromosome 1B of Kenong199 (KN199) and chromosomes 1A, 1B and 1D of Chinese spring (CS).
**Figure S2** Morphological characters of the *pUbi:tae‐MIR172* transgenic bread wheat plants.
**Figure S3** The coding sequence of Q from bread wheat cultivar KN199.
**Figure S4** The domestication‐related traits of the spikes of the *pUbi:tae‐MIR172* transgenic bread wheat lines.
**Figure S5** The coding sequence of *TaTPL* from the A genome of bread wheat cultivar KN199.
**Figure S6** The coding sequence of *TaTPL* from the D genome of KN199.
**Table S1** Prediction of putative target genes of tae‐miR172 in bread wheat.
**Table S2** Constructs used in this study.
**Table S3** Primers used in this study.
