# Supplementary material for: Functional regulation of Q by microRNA172 and transcriptional co‐repressor TOPLESS in controlling bread wheat spikelet density
Source: Plant Biotechnol J. 2017 Aug 9;16(2):495–506. doi: 10.1111/pbi.12790 (PMC5787848; doi:10.1111/pbi.12790)
Supplement: Supplementary file 1 — Figure S1 Sequence alignment of tae‐MIR172 precursors from chromosome 1B of Kenong199 (KN199) and chromosomes 1A, 1B and 1D of Chinese spring (CS). Figure S2 Morphological characters of the pUbi:tae‐MIR172 transgenic bread wheat plants. Figure S3 The coding sequence of Q from bread wheat cultivar KN199. Figure S4 The domestication‐related traits of the spikes of the pUbi:tae‐MIR172 transgenic bread wheat lines. Figure S5 The coding sequence of TaTPL from the A genome of bread wheat cultivar KN199. Figure S6 The coding sequence of TaTPL from the D genome of KN199. Table S1 Prediction of putative target genes of tae‐miR172 in bread wheat. Table S2 Constructs used in this study. Table S3 Primers used in this study. [file PBI-16-495-s001.pdf]

Supporting Information

Figure S1

tae-MIR172

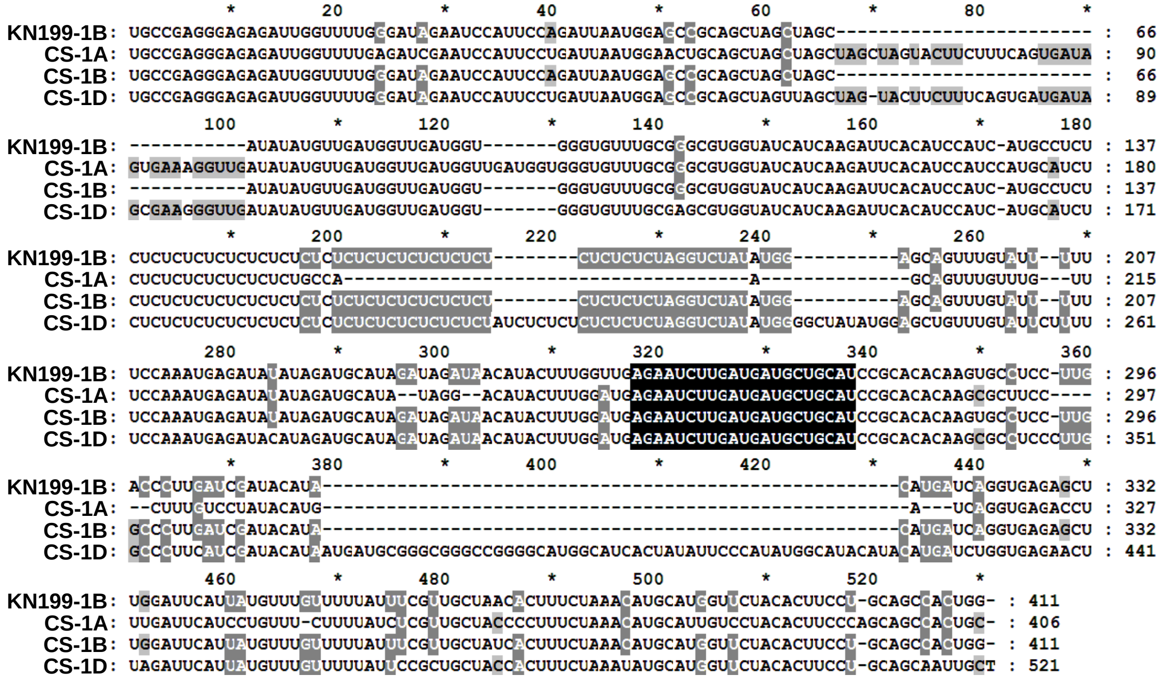

Figure S1. Sequence alignment of *tae-MIR172* precursors from chromosome 1B of Kenong199 (KN199) and chromosomes 1A, 1B and 1D of Chinese spring (CS). The mature tae-miR172 sequences are represented by black shade boxes.

Figure S2

(a)

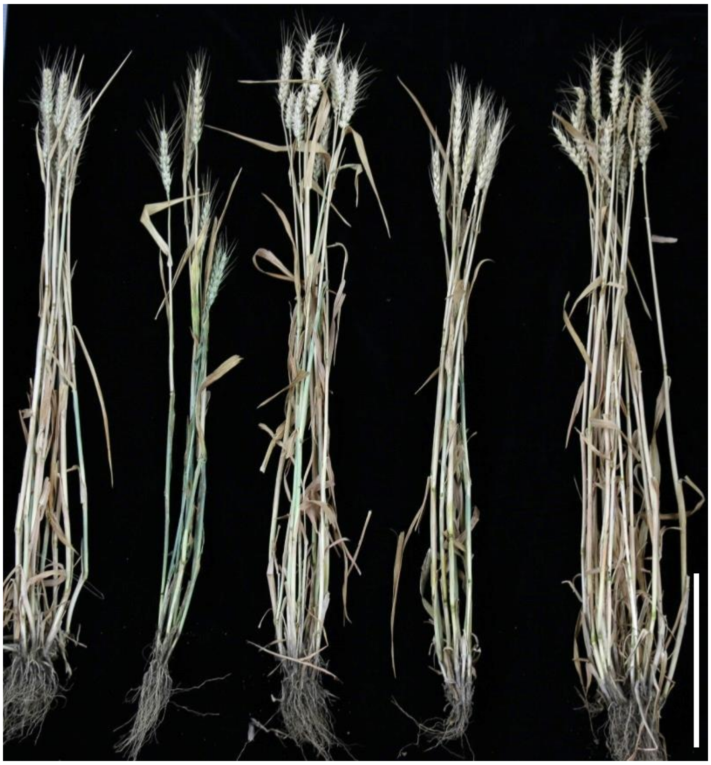

#1 #3 #4 #5

KN199

pUbi:tae-MIR172

(b)

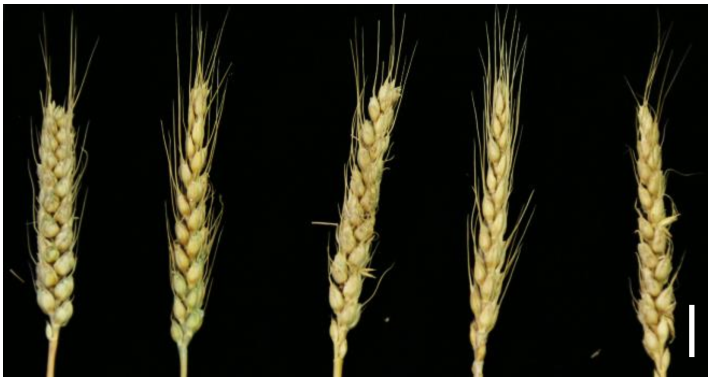

#1 #3 #4 #5

KN199

pUbi:tae-MIR172

Figure S2. Morphological characters of the *pUbi:tae-MIR172* transgenic bread wheat plants. (a) Whole plant view of different *pUbi:tae-MIR172* transgenic lines. Bar = 20 cm. (b) The spike phenotypes of the *pUbi:tae-MIR172* transgenic lines. Bar = 2 cm. #1, #3, #4 and #5 represent independent transgenic lines.

Figure S3

5’-
ATGGTGCTGGATCTCAATGTGGAGTCGCCGGCGGACTCGGGCACGTCCAGCTCCTCCG
TGCTCAACTCCGCGGACGCCGGTGGCGGCGGCTTCCGGTTCGGCCTGCTCGGGAGCC
CTGATGATGACGACTGCTCCGGCGAGCCGGCGCCGGTCGGGCCCGGGTTCGTCACGA
GGCAGCTCTTCCCCGCGTCGCCGCCCGGGCACGCGGGCGCGCCCGGGGTGACGATG
GGGCAGCAGGCCCCGGCGCCTGCGCCGATGGCGCCCGTGTGGCAGCCGCGGCGCGC
CGAGGAGCTCCTCGTGGCGCAGCGGATGGCGCCCGCGAAGAAGACGCGGCGGGGCC
CGAGGTCGCGCAGCTCGCAGTACAGGGGCGTCACCTTCTACCGCAGGACCGGCCGGT
GGGAGTCGCACATCTGGGATTGCGGGAAGCAGGTCTACTTGGGTGGTTTCGACACTGC
GCACGCGGCCGCAAGGGCCTACGATCGCGCGGCGATCAAGTTCCGGGGGCTGGAGGC
CGACATCAACTTCAATCTGAGCGACTACGAGGAGGATTTGAAGCAGATGAGGAACTGGA
CCAAGGAGGAGTTCGTGCACATCCTCCGCCGCCAGAGCACGGGGTTCGCCAGGGGGA
GCTCCAAGTACCGCGGCGTCACGCTCCACAAGTGCGGCCGCTGGGAGGCAAGGATGG
GCCAGCTGCTCGGCAAGAAGTACATATATCTGGGCCTCTTTGACAGCGAAGTTGAAGCT
GCAAGGGCGTACGACAGGGCGGCGATTCGCTTCAATGGGAGGGAAGCTGTGACTAACT
TTGAGAGCAGCTCCTACAATGGGGATGCTCCACCCGACGCCGAAAATGAGGCAATTGTT
GATGCTGATGCTCTTGACTTGGATCTGCGGATGTCGCAACCCACCGCGCACGATCCCA
AGAGGGACAACATCATCGCCGGCCTTCAGTTAACTTTTGATTCCCCTGAATCGTCAACC
ACAATGATCTCTTCTCAGCCAATGAGCTCATCTTCGTCCCAGTGGCCTGTGCATCAACAT
GGCACGGCAGTAGCACCTCAGCAGCACCAGCGTTTGTACCCATCTGCTTGTCATGGCTT
CTACCCGAACGTACAGGTGCAGGTGCAGGAGAGGCCCATGGAGGCAAGGCCCCCTGA
GCAGCCGTCGTCCTTCCCCGGCTGGGGGTGGCAAGCGCAAGCCATGCCGCCGGGCTC
CTCCCACTCGCCGTTGCTTTACG**CTGCAGCATCATCAGGATTTT**CTACCGCCGCCGCC
GGCGCGAACCTCGCCCCGCCGCCGCCGTACCCGGACCACCACCGGTTCTACTTCCCC
CGCCCGCCGGACAACTGA-3’

Figure S3 The coding sequence of *Q* from bread wheat cultivar KN199. The miR172-targeting site is highlighted in red.

Figure S4

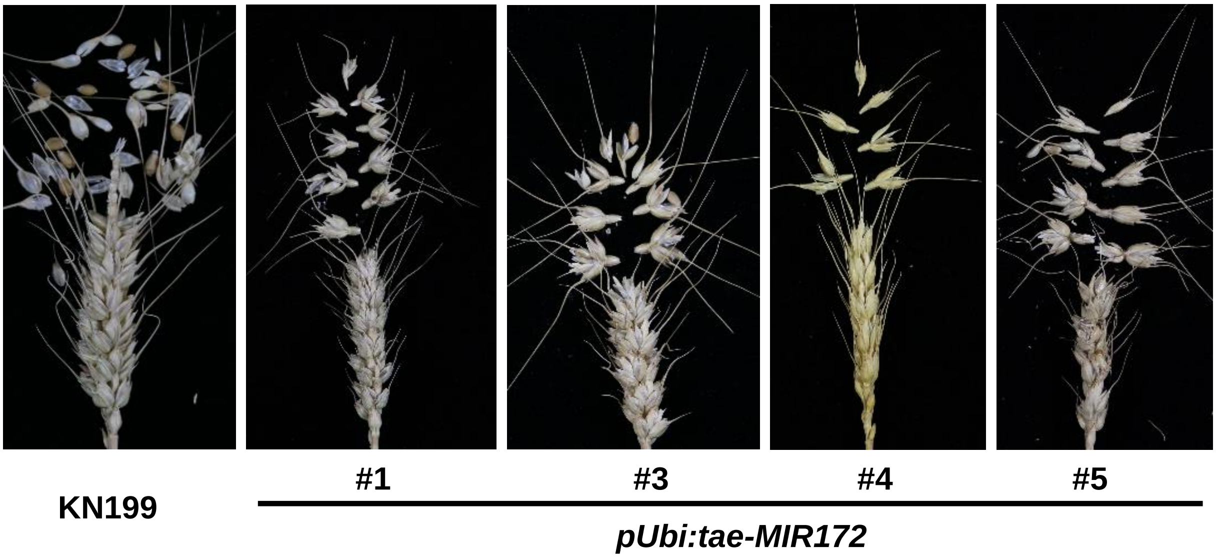

Figure S4 The domestication-related traits of the spikes of *pUbi:tae-MIR172* transgenic lines.

Figure S5

5’-
ATGTCTTCTCTCAGCCGGGAGCTCGTCTTCCTCATCCTGCAGTTCCTCGATGAGGAGAAGTTCAAGG
AGACCGTCCACAAGCTTGAGCAGGAGTCTGGCTTCTACTTCAACATGAAGTACTTCGAGGATGAGGT
GATCAATGGCAATTGGGATGAGGTGGAGCGCTACCTCGGGGGCTTCACCAAAGTCGACGACAACC
GCTACTCGATGAAGATATTCTTTGAGATCCGCAAGCAGAAGTATCTCGAGGCCCTCGACAAGCATGA
TCGGTCCAAGGCGGTTGAAATCTTGGTCAAGGACTTGAAGGTGTTTGCATCCTTTAATGAGGAGCTG
TTTAAGGAGATCACACAGCTATTGACTCTGGAGAACTTCAGGGAGAATGAGCAGCTCTCCAAGTATG
GTGATACAAAATCTGCAAGAGCAATAATGCTTGTTGAGCTGAAGAAGCTGATCGAAGCTAACCCCTT
ATTCCGTGACAAGCTTCAGTTTCCCAATCTGAAGAGTTCTAGATTACGGACACTTATCAATCAGAGCT
TAAACTGGCAGCACCAGCTTTGCAAAAATCCCAGGCCTAATCCTGACATCAAGACCCTCTTTGTTGA
TCATTCTTGTGGACAACCAAATGGTGCACGTGCTCCATCACCAGCAAACAATCCACTACTTGGATCT
ATACCTAAACCTGGTGGTTTCCCCCAATTGGGCGCCCATGGACCCTTTCAACCTGCACCAACACCTG
TCGCACCTCTGGCCGGTTGGATGTCAAACCCTCCAGCAGTAACACATCCTGCTGTTTCTGGAGGTG
CTATTGGATTTGGTACTCCTACGAATCCTGCTGCTATGTTGAAGCATCCTAGGACACCCTCGGCTGC
TAACCCTTCTATGGACTATCCATCTGGAGATTCTGATCATGTCAGTAAGAGACCCAGGCCAGTTGGG
TTGTCTGAGGAGGTGAATCTTCCGGTGAATATGATGCCAGTAACTTATCCGCAGAGCCATAGCTACC
CGCAAGATGATTTCCATAAAGCTGTCGCACGGACATTGAGTCAAGGATCAGCTCCAATGAGCATGGA
TTTCCATCCAGTTCAACAAACTCTTCTTCTTGTTGGTACCAATGTTGGTGACATTGGATTGTGGGATG
TTGGCACCAAGGAGAGACTTGTTGTAAGAAACTTCAAGGTTTGGGAGCTTGGGAAATGTTCTATGGC
CCTCCAGGCAGCACTGGTTAAGGATCCTACCGTGTCAGTTAATCGCATAATTTGGAGTCCTGATGGA
ACCTTGTTTGGTGTTGCTTATTCAAGGCATATAGTACAGATTTATTCTTATCATGGTGGCGATGATATT
AGGCAACACTTGGAGATTGATGCACATGTTGGTGGCGTAAATGACATTGCATTCGCCCATCCAAATA
AGCAGCTGTGTATAATTACCTGTGGAGACGATAAGACGATAAAGGTGTGGGAGGCCACTAGTGGAA
CAAAACAATTTACCTTCGAAGGTCATGAAGCTCCTGTCTATTCTGTTTGTCCACATTACAAGGAAAAT
ATTCAGTTCATCTTTTCAACTGCTTTAGATGGAAAGATAAAGGCATGGCTGTATGATAATCTTGGGTC
CAGAGTTGACTATGATGCACCAGGTCATTGGTGCACTACGATGGCATATAGTGCGGATGGTTCAAGA
TTGTTTTCTTGCGGGACCAGCAAGGATGGTGAATCACACCTGGTGGAATGGAATGAGAGTGAAGGT
GCGGTGAAGAGAACATACCAGGGATTTCGTAAGCGATCTATGGGTGTTGTACAATTCGATACGACGC
GAAATAGGTTTTTGGCTGCTGGAGATGAATTCGTGATAAAGATTTGGGACATGGACAACACTAGTCT
TCTGACTACCATTGAAGCTGATGGTGGCCTACCTGCAAGCCCGCGTATCCGCTTTAACAAGGAGGG
TACTCTGTTGGCAGTCTCTACTGTTGATAATGGTATCAAAGTCTTAGCAAATGCTGATGGTGTTCGTT
TATTGCGCACACTGGAAAATCGTTCTTTTGATGCTTCACGTAGTGCATCTGAGACTGTAACAAAGCCT
CTAATAAACCCATTGACTGCTGCTGCTGCGGCAGCCGCCGCCGCTGCCGCCGCTGCAACCAGTTCT
GGAACTGCTGCTCCATCACCTATAACTGCAATGAACGGGGACAACAGAAGCATGGTTGATGTAAAAC
CTAGAATAGCTGATGAGTCAATGGATAAGTCAAAGGTCTGGAAGCTTATGGAGATAACTGATACAGC
TCAGTGCAGATCATTAAAACTAGGTGATAGTATTAGGACAGCTAAGATCTCGAGACTGATCTACACAA
ATTCAGGTGTTGCTATCTTGGCTTTAGCTTCAAATGCCGTCCATCTACTGTGGAAATGGCCACGTAAT
GAACGAAATTCAACTGGAAAGGCTACTGCTAGTGTTTCTCCTCAATTATGGCAACCTCCAAGTGGCA
TTCTCATGACTAATGACACAATTGACAATAGTCCTGATGAGGCCGTTCACTGCTTTGCTTTGTCAAAG
AATGATTCATATGTCATGTCAGCTTCAGGAGGGAAAATCTCTCTATTCAACATGATGACTTTTAAGAC
GATGACGACGTTTATGCCTCCGCCACCAGCAGCTACTTTCCTCGCATTCCATCCTCAAGATAACAAC
ATAATTGCAATTGGAATGGATGATTCAACCATCCAAATTTACAACGTTCGGATTGATGAGGTCAAAAG
TAAACTTAGAGGGCACTCTAAGAAAATTACAGGTCTTGCCTTTTCAAATGTGTTAAATGTTTTGGTATC
CTCTGGAGCTGATGCACAGATATGTGTTTGGAACACTGATGGGTGGGAGAGGCAAAGGAGCAGATT
TTTGCAGATACCATCTGGTCGCCCAACGTCCAACATCTTAGACACACGAGTTCAGTTCCACCAAGAT
CAACAGCATTGTCTTGTTGTCCATGAGACCCAGATTGCCATCTATGATGCCTCAAAATTAGAACCCGT
GAAGCAGTGGCCTCCTCGGGAGACATCTGCTCCTCCAATAACGCATGCTACATTCTCATGTGATAGT
CAACTGATTTATGCAAGCTTTCTAGATGCCACTGTCTGCATATTTAGCGCATCAAGTTTGAGACTTCA
ATGCCGAATTCTTCCAGCTTCTTATCTTCCTCAAAATATCAGTTCAAATGTTCATCCAGTTGTGGTTGC
GGCACATCCTTCGGAAGCAAATCAGTTTGCTCTAGGCCTTACCGATGGCAGCGTTTATGTCATGGAA
CCGTTGGAATCTGAGAGAAAGTGGGGAATTCCTCCACCAGCGGAGAATGGATCGACGAGCAACATG
TCCACACCTCCTAATGGAGCTTCGAGTTCTGATCAACCAGAAAGATAA-3’

Figure S5 The coding sequence of *TaTPL* from the A genome of bread wheat cultivar KN199.

Figure S6

5'-
ATGTCTTCTCTCAGCCGGGAGCTCGTCTTCCTCATCCTGCAGTTCCTCGATGAGGAGAAGTTCAAGG
AGACCGTCCACAAGCTCGAGCAGGAGTCTGGTTTCTACTTCAACATGAAGTACTTTGAGGACGAGGT
GATCAATGGGAATTGGGATGAGGTGGAGCGCTACCTCGGGGGCTTCACCAAAGTCGACGACAACC
GCTACTCGATGAAGATATTCTTTGAGATCCGCAAGCAGAAGTATCTCGAGGCCCTCGATAAGCATGA
TCGGTCCAAGGCGGTTGAAATCTTGGTCAAGGACTTGAAGGTGTTTGCATCCTTTAACGAGGAGCTG
TTTAAGGAGATAACACAGCTATTGACTCTGGAGAACTTCAGGGAGAATGAGCAGCTCTCCAAGTATG
GTGATACAAAATCTGCAAGAGCAATAATGCTTGTTGAGCTGAAGAAGCTGATCGAAGCTAACCCCTT
ATTCCGTGATAAGCTCCAGTTTCCCAATCTGAAGAGCTCTAGATTACGGACACTTATCAACCAGAGC
TTAAACTGGCAGCACCAGCTTTGCAAAAATCCTAGGCCTAATCCTGACATCAAGACCCTCTTTGTTGA
TCATTCTTGTGGACAACCAAATGGTGCACGTGCTCCATCACCAGCAAACAATCCACTACTTGGATCT
ATACCTAAACCTGGTGGTTTCCCCCAATTGGGCGCCCATGGACCCTTTCAACCTGCACCAACACCC
GTCGCACCTCTGGCCGGTTGGATGTCAAACCCTCCAGCAGTAACGCATCCTGCTGTTTCTGGAGGT
GCTATTGGATTTGGTACTCCTACGAATCCTGCTGCTATGTTGAAGCATCCTAGGACACCCTCGGCTG
CTAACCCTTCTATGGATTATCCATCTGGAGATTCTGATCATGTCAGTAAGAGACCTAGGCCAGTTGG
GTTGTCTGAGGAGGTGAATCTTCCGGTGAATATGATGCCAGTAACTTATCCGCAGAGCCATAGCTAC
CCGCAAGATGATTTCCATAAAGCTGTCGCACGGACATTGAGTCAAGGATCAGCTCCAATGAGCATGG
ATTTCCATCCAGTTCAACAAACTCTTCTTCTTGTTGGTACCAATGTTGGTGACATTGGATTGTGGGAT
GTTGGCACCAAGGAGAGACTTGTTGTAAGAAACTTCAAGGTTTGGGAGCTTGGGAAATGTTCTATGG
CCCTCCAGGCTGCACTGGTTAAGGATCCTACCGTGTCAGTTAATCGCATAATATGGAGTCCTGATGG
AACCTTGTTTGGTGTTGCTTATTCAAGGCATATAGTACAGATTTATTCTTATCATGGTGGCGATGATAT
TAGGCAACACTTGGAGATTGATGCACATGTTGGTGGCGTAAATGACATTGCATTCGCCCATCCAAAT
AAGCAGCTCTGTATAATTACCTGTGGAGACGATAAGACGATAAAGGTGTGGGAGGCCACCAGTGGA
ACAAAACAATTTACCTTTGAAGGTCATGAAGCTCCTGTCTATTCTGTTTGTCCACATTACAAGGAAAA
TATTCAGTTCATCTTTTCAACTGCTTTAGATGGAAAGATAAAGGCATGGCTCTATGATAATCTTGGGT
CCAGAGTTGACTATGATGCACCAGGTCATTGGTGCACTACGATGGCATATAGTGCGGATGGGTCAA
GATTATTTTCTTGTGGGACCAGCAAGGATGGTGAATCACACCTGGTGGAATGGAATGAGAGTGAAG
GTGCGGTGAAGAGAACATACCAGGGATTTCGTAAGCGATCGATGGGTGTTGTACAATTTGATACGAC
GCGAAATAGGTTTTTGGCTGCTGGAGATGAGTTCGTGATAAAGATTTGGGACATGGACAACACTAGT
CTTCTGACTACCATTGAAGCTGATGGTGGCCTACCTGCAAGCCCGCGTATCCGCTTTAACAAGGAG
GGTACTCTGTTGGCAGTCTCTACTGTTGATAATGGTATCAAAGTCTTAGCAAATGCTGATGGTGTTCG
TTTATTGCGCACACTGGAAAATCGTTCTTTTGATGCTTCTCGTAGTGCATCTGAGACTGTAACAAAGC
CTCTTATAAACCCATTGACTGCTGCTGCCGCGGCAGCTGCCGCCGCCGCCGCTGCAACCAGTTCTG
GAACTGCTGCTCCATCATCTATAACTGCAATGAATGGGGACAACAGAAGCATGGTTGATGTAAAACC
TAGAATAGCTGATGAGTCAATGGATAAGTCAAAGGTCTGGAAGCTTATGGAGATAACTGATACAGCT
CAGTGCAGATCACTAAAACTAGGTGATAGTATTAGGACAGCTAAGATCTCGAGACTGATCTACACAA
ATTCAGGTGTTGCTATCTTGGCTTTAGCTTCAAATGCCGTCCATCTACTGTGGAAATGGCCACGTAAT
GAACGAAATTCAACTGGAAAGGCTACTGCTAGTGTTTCTCCTCAATTATGGCAACCTCCAAGTGGCA
TTCTCATGACTAATGACACAATTGACAATAGTCCTGATGAGGCCGTTCACTGCTTTGCTTTGTCAAAG
AATGATTCATATGTCATGTCAGCTTCAGGAGGGAAAATCTCTCTATTCAACATGATGACTTTTAAGAC
GATGACAACGTTTATGCCTCCACCACCAGCAGCTACTTTCCTAGCATTCCATCCTCAAGATAACAACA
TAATTGCAATTGGAATGGATGATTCAACCATCCAAATTTACAACGTTCGGATTGATGAGGTCAAAAGC
AAACTTAGAGGGCACTCTAAGAAAATTACAGGTCTCGCCTTTTCAAATGTGTTAAATGTTTTGGTATC
CTCTGGAGCTGATGCACAGATATGTGTTTGGAACACTGATGGGTGGGAGAGGCAAAGGAGCAGATT
TTTGCAGATACCGTCTGGCCGCCCAACGTCCAACATCTTAGACACACGAGTTCAGTTCCACCAAGAT
CAACAGCATTGTCTTGTTGTCCATGAGACCCAGATTGCCATCTATGATGCCTCAAAACTAGAACCCG
TGAAGCAGTGGCCTCCTCGGGAGACCTCTGCTCCTCCAATAACGCATGCTACATTCTCATGTGATAG
TCAACTGATTTATGCAAGCTTTCTGGACGCCACTGTCTGCATATTTAGCGCATCAAGTTTGAGACTCC
AATGCCGAATTCTTCCAGCTTCTTATCTTCCTCAAAATATCAGTTCAAATGTTCATCCAGTTGTGGTTG
CGGCACATCCTTCGGAGGCAAATCAGTTTGCTCTAGGCCTTACTGATGGCAGTGTTTATGTCATGGA
ACCGTTGGAATCTGAGAGAAAGTGGGGAATTCCTCCACCAGCGGAGAATGGATCGACGAGCAACAT
GTCCACACCTCCTAATGGAGCTTCGAGTTCTGATCAACCAGAAAGATAA-3'

Figure S6 The coding sequence of *TaTPL* from the D genome of KN199.

Table S1. Prediction of putative target genes of tae-miR172 in bread wheat. UPE, maximum energy to un-pair.

Target accession number	E-value	UPE	Target aligned fragment	Target description
CA609410	0.5	12.522	UGCAGCAUCAU CAGGAUUCU	weakly similar to UniRef100_Q0DL60 Cluster: Os05g0121600 protein; n=1; Oryza sativa Japonica Group|Rep: Os05g0121600 protein - Oryza sativa subsp. japonica (Rice), partial (11%)
CA626451	0.5	21.238	UGCAGCAUCAU CAGGAUUCU	UniRef100_A6NAX8 Cluster: AP2 domain transcription factor; n=1; Zea mays|Rep: AP2 domain transcription factor - Zea mays (Maize), partial (25%)
CA652598	0.5	13.558	UGCAGCAUCAU CAGGAUUCU	similar to UniRef100_Q0DL60 Cluster: Os05g0121600 protein; n=1; Oryza sativa Japonica Group|Rep: Os05g0121600 protein - Oryza sativa subsp. japonica (Rice), partial (10%)
CA486144	0.5	11.277	UGCAGCAUCAU CAGGAUUCU	similar to UniRef100_Q2TQ34 Cluster: Transcription factor AP2D23-like; n=2; Oryza sativa|Rep: Transcription factor AP2D23-like - Oryza sativa subsp. japonica (Rice), partial (18%)
TC438982	0.5	13.048	UGCAGCAUCAU CAGGAUUCU	similar to UniRef100_Q0DL60 Cluster: Os05g0121600 protein; n=1; Oryza sativa Japonica Group|Rep: Os05g0121600 protein - Oryza sativa subsp. japonica (Rice), partial (9%)
TC427232	0.5	12.864	UGCAGCAUCAU CAGGAUUCU	weakly similar to UniRef100_Q0DL60 Cluster: Os05g0121600 protein; n=1; Oryza sativa Japonica Group|Rep: Os05g0121600 protein - Oryza sativa subsp. japonica (Rice), partial (10%)
TC392280	0.5	13.048	UGCAGCAUCAU CAGGAUUCU	weakly similar to UniRef100_Q0DL60 Cluster: Os05g0121600 protein; n=1; Oryza sativa Japonica Group|Rep: Os05g0121600 protein - Oryza sativa subsp. japonica (Rice), partial (18%)
TC401083	0.5	13.215	UGCAGCAUCAU CAGGAUUCU	similar to UniRef100_Q2TQ34 Cluster: Transcription factor AP2D23-like; n=2; Oryza sativa|Rep: Transcription factor AP2D23-like - Oryza sativa subsp. japonica (Rice), partial (62%)
TC374382	0.5	13.215	UGCAGCAUCAU CAGGAUUCU	similar to UniRef100_Q2TQ34 Cluster: Transcription factor AP2D23-like; n=2; Oryza sativa|Rep: Transcription factor AP2D23-like - Oryza sativa subsp. japonica (Rice), partial (76%)
CA648892	1	16.893	UGCAGCAUCAU CAGGAUUUU	UniRef100_Q5Y165 Cluster: Floral homeotic protein; n=3; Triticum|Rep: Floral homeotic protein - Triticum turgidum subsp. dicoccon, partial (13%)
TC405024	1	17.371	UGCAGCAUCAU CAGGAUUUU	UniRef100_Q5Y165 Cluster: Floral homeotic protein; n=3; Triticum|Rep: Floral homeotic protein - Triticum turgidum subsp. dicoccon, partial (15%)
TC400547	1	17.371	UGCAGCAUCAU CAGGAUUUU	UniRef100_Q5Y383 Cluster: Floral homeotic protein; n=1; Triticum turgidum subsp. carthlicum|Rep: Floral homeotic protein - Triticum turgidum subsp. carthlicum, partial (49%)
TC381895	1	17.371	UGCAGCAUCAU CAGGAUUUU	UniRef100_Q5Y383 Cluster: Floral homeotic protein; n=1; Triticum turgidum subsp. carthlicum|Rep: Floral homeotic protein - Triticum turgidum subsp. carthlicum, partial (36%)
TC368660	1	17.371	UGCAGCAUCAU CAGGAUUUU	UniRef100_Q5Y386 Cluster: Floral homeotic protein; n=5; Triticum|Rep: Floral homeotic protein - Triticum aestivum (Wheat), complete
NP9350139	1.5	17.562	GGCAGCAUCAU CAGGAUUCU	GB|AY714341.1|AAU94918.1 floral homeotic protein;AP2-like transcription factor; Q protein
GH725141	2.5	20.811	GGCAGCAUCAU CAAGAUCCU	similar to UniRef100_Q2PYW7 Cluster: Succinyl CoA ligase beta subunit-like protein; n=1; Solanum tuberosum|Rep: Succinyl CoA ligase beta subunit-like protein - Solanum tuberosum (Potato), partial (11%)
TC404380	2.5	18.186	UGCAGCAUCAU CACGAUUCC	similar to UniRef100_Q0J9J3 Cluster: Os04g0649100 protein; n=1; Oryza sativa Japonica Group|Rep: Os04g0649100 protein - Oryza sativa subsp. japonica (Rice), partial (26%)
CA644318	2.5	20.726	AAGCUGCAUCA UCAAGAUUUU	
CA727182	3	11.11	AGUAGUAUUAU CAAGAUUUU	
TC438028	3	22.883	AUGCGCAAUCA UCAAGAUUUU	
TC420199	3	21.72	UGUGGUAACAU UAAGAUUCU	homologue to UniRef100_Q7XYC9 Cluster: 60s ribosomal protein L21; n=1; Triticum aestivum|Rep: 60s ribosomal protein L21 - Triticum aestivum (Wheat), partial (97%)
DR731547	3	14.115	UUCAGCCUUAU UAAGAUUCU	

Table S2. Constructs used in this study.

Construct name	Vector	Description
pUbi:tae-MIR172	*pUbi:cas*	For gene tansformation
p35S:tae-MIR172	*pGreen0800*	For transient expression assay in *N. benthamiana*
p35S:Q-Myc	*PGWB17*	For transient expression assay in *N. benthamiana*
p35S:mQ-Myc	*PGWB17*	For transient expression assay in *N. benthamiana*
p35S:Q-GFP	*PGWB5*	For subcellular localization analysis in *N. benthamiana*
BD-Q	*pGBKT7*	For luciferase transient expression assay
p35S:GAL4-BD-Q	*P2GW7*	For luciferase transient expression assay
AD-Q	*pGADT7*	For Y2H assay
BD-TaTPL	*pGBKT7*	For Y2H assay
nYFP-TaTPL	*pEarly201*	For BiFC assay in *N. benthamiana*
nYFP-Q	*pEarly202*	For BiFC assay in *N. benthamiana*
nLUC-Q	*p1300-35S-nLUC*	For LCI assay in *N. benthamiana*
nlUC-Q^{mEAR1}	*p1300-35S-nLUC*	For LCI assay in *N. benthamiana*
nLUC-Q-N	*p1300-35S-nLUC*	For LCI assay in *N. benthamiana*
nLUC-Q-M	*p1300-35S-nLUC*	For LCI assay in *N. benthamiana*
nLUC-Q-C	*p1300-35S-nLUC*	For LCI assay in *N. benthamiana*
cLUC-TaTPL	*p1300-35S-cLUC*	For LCI assay in *N. benthamiana*
cLUC-TaTPL-N	*p1300-35S-cLUC*	For LCI assay in *N. benthamiana*
cLUC-TaTPL-NΔCTHL	*p1300-35S-cLUC*	For LCI assay in *N. benthamiana*
cLUC-TaTPL-C	*p1300-35S-cLUC*	For LCI assay in *N. benthamiana*

Table S3. Primers used in this study.

Primer name	Sequence (5'-3')
pUbi:tae-MIR172-F	CGCGGATCCTGCCGAGGGAGAGATTGGTT
pUbi:tae-MIR172-R	CGGGGTACCCCAGTGGCTGCAGGAAGTGTAG
Q-F1	AGGAGTGCGTTTTATTCGGTCCCGG
Q-R1	CCACCGCCGTCTGGTCACAACG
Q-F	ATGGTGCTGGATCTCAATGTGGAGTCGC
Q-R(-stop)	GTTGTCCGGCGGGCGGGGGAA
SOE-mQ-F	GTCTTATTGCCCTGCACTGCCGTGCCG
SOE-mQ-R	GCGCTACGGTTGACGGCACGGCAGTGCA
35S:tae-MIR172-F	CTTCGGTACCTGCCGAGGGAGAGATTGGTT
35S:tae-MIR172-R	CTTCGGATCCCCAGTGGCTGCAGGAAGTGTAG
TaTPL-outer-F	CAGGCTAGGTTTACGGCCAGG
TaTPL-outer-R	CTGAGATGGATACCTTGGTTAGTG
TaTPL-F	ATGTCTTCTCTCAGCCGGGAGC
TaTPL-R	TTATCTTTCTGGTTGATCAGAACTCGA
TaTPL-R(-stop)	TCTTTCTGGTTGATCAGAACTCGA
AD-Q-F	GGAATTCCATATGATGGTGCTGGATCTCAATGTGGAG
AD-Q-R(-stop)	CGGAATTCGTTGTCCGGCGGGCGGGGGAA
BD-TaTPL-F	AGGAGGACCTGCATATGATGTCTTCTCTCAGCCGGGAGC
BD-TaTPL-R	ACGGATCCCCGGGAATTCTCTTTCTGGTTGATCAGAACTCGA
TaTPL-NΔCTHL-F	GTTTCTACTTCCGGTTGAAATCTTGGTCAAGGACTT
TaTPL-NΔCTHL-R	ATTTCAACCGGAAGTAGAAACCAGACTCCTGCTCG
nLUC-Q-F	GGGGTACCATGGTGCTGGATCTCAATGTGGAGTCG
nLUC-Q-R	GCGTCGACGTTGTCCGGCGGGCGGGG
nLUC-Q-N-R	GCGTCGACCCCATTGTAGGAGCTGCTCTCA
nLUC-Q-C-F	GGGGTACCGATGCTCCACCCGACGCC
Q^{mEAR1}-F	ATGGTGGCCGCTGCCGCTGTGGAGTCGCCGGCGGACTCG
nLUC-Q^{mEAR1}-F	GGGGTACCATGGTGGCCGCTGCCGCTGTG
cLUC-TaTPL-F	ACGCGTCCCGGGGCGGTACCATGTCTTCTCTCAGCCGGGAGC
cLUC-TaTPL-R	GTTGCTGCAGGTCGACTTATCTTTCTGGTTGATCAGAACTCGA
cLUC-TaTPL-N-R	GTTGCTGCAGGTCGACTTAGAAATCATCTTGCGGGTAGCTATG
cLUC-TaTPL-C-F	ACGCGTCCCGGGGCGGTACCCATAAAGCTGTCGCACGGAC
BD-Q-F	AGGAGGACCTGCATATGATGGTGCTGGATCTCAATGTGGAG
BD-Q-R(-stop)	ACGGATCCCCGGGAATTCGTTGTCCGGCGGGCGGGGGAA
BD-F	ATGAAGCTACTGTCTTCTATCGAACAAGC
5'RACE-Q-outer-R	TCGACCGCGCGCACCCACCGC
5'RACE-Q-inner-R	CACCCACCGCCGTCTGGTCACAA
tae-miR172-RT	GTCGTATCCAGTGCAGGGTCCGAGGTATTCGCACTGGATACGACAT GCAG
Real-Q-F	GCACCTCAGCAGCACCAGCGTTT
Real-Q-R	GCGGGCGGGGGAAGTAGAACC
Real-tae-miR172-F	CGGGCTAGAATCTTGATGATGC
